# Donor human milk practice in Indonesia: a media content analysis

**DOI:** 10.3389/fnut.2024.1442864

**Published:** 2024-09-18

**Authors:** Andini Pramono, Alvia Hikmawati

**Affiliations:** ^1^Department of Health Economics, Wellbeing and Society, National Centre for Epidemiology and Population Health, Australian National University, Canberra, ACT, Australia; ^2^Research Division, Indonesian Breastfeeding Mother Association, Surabaya, Jakarta, Indonesia

**Keywords:** breastfeeding, donor milk, breastmilk, infant feeding, content analysis

## Abstract

**Introduction:**

Donor human milk (DHM) is recommended as the second-best alternative form of supplementation when a mother is unable to breastfeed directly. However, little is known about the experience of mothers and families in the communities regarding accessing and donating expressed breastmilk in Indonesia. This study aimed to identify the experience related to donor human milk in the society in Indonesia.

**Method:**

A search was conducted through six main online news portals. The keywords used included “donor human milk,” “expressed breastmilk,” and “wet nursing” in the Indonesian language, Bahasa Indonesia. A total of 107 articles were found, but only 20 articles were included for analysis using a qualitative media content analysis approach.

**Results:**

In the study, the following five themes were identified: (1) the whys and wherefores of donor human milk, (2) national and religious-based regulations, (3) recommendations from authorized organizations, healthcare professionals, and Islamic scholars, (4) the negative impact from the lack of national regulations, and (5) contradictory feelings among mothers.

**Conclusion:**

With the lack of detailed information on how to access or donate expressed human milk and the absence of a human milk bank in place, informal human milk sharing is inevitably occurring in the community. This has also raised concerns among authorized organizations, healthcare professionals, and Islamic scholars. Consequently, mothers, both donors and recipients, experienced negative impacts, which included contradictory feelings. Engaging with Islamic scholars and healthcare professionals to develop clear guidelines and regulations to enable mothers' and families' access and/or make contributions to DHM in a safe and accountable way is critical to prevent further problems from occurring in Indonesian society.

## 1 Introduction

Breastfeeding exclusively for the first 6 months of an infant's life is recommended as the gold standard by the World Health Organization (WHO) as it provides all the nutrients a baby needs (1). Along with complementary nutritious food, breastmilk continues to provide up to half or more of a child's nutritional needs during the second half of the first year and up to one-third of the needs during the second year of life (1). However, when mothers are unable to breastfeed their babies due to medical conditions or when babies and their mothers are separated by sickness, death, or an emergency, the best alternative is to get expressed breastmilk from the infant's own mother (2, 3). If it is not possible, then donor human milk (DHM) or wet nursing is the next recommendation, and a breastmilk substitute fed with a cup is the last resort (2, 3).

Indonesia is a developing country in Southeast Asia, with 277 million citizens and 4.46 million live births estimated in 2023 (4). However, only 37.3% of babies under 6 months old were exclusively breastfed in 2018 (5). The systemic barriers to optimal breastfeeding discussed in the Lancet papers (6–8) are occurring in Indonesia. Although the Indonesian government protects, supports, and promotes breastfeeding and has adopted the 1989 Ten Steps to Successful Breastfeeding (Ten Steps) (9) into its national law (10), the WHO International Code of Marketing of Breastmilk Substitute (WHO Code) and the subsequent World Health Assembly (WHA) resolutions (11) have not been fully adopted in the national legal framework. The WHO Code and the relevant WHA resolution are essential to ensure that parents and other caregivers are protected from inappropriate and misleading information (12). Therefore, it is included in the 2018 Ten Steps (9), but it has not been adopted in the Indonesian national regulations. This is one of the key factors why breastfeeding protection is still lacking in Indonesia (13). Violations of the Code by health workers, breastmilk substitute companies, and their representatives were also found in provinces in Indonesia (13, 14).

The marketing of breastmilk substitutes has also been reaching the digital world through social media, such as Facebook, Instagram, and TikTok (15). Indonesian mothers and families often access information through the Internet (16), which includes seeking information about breastfeeding support and access to donor human milk (DHM). At the time of writing this article, there were no data on the existence of any human milk bank in Indonesia; thus, the practice of informal milk sharing, including through the Internet, inevitably occurs. Little is known about the experience of mothers and families in the communities regarding accessing and donating expressed breastmilk or the practice of wet nursing in Indonesia. This study aimed to identify the experience related to DHM in Indonesian society using media content analysis.

## 2 Methods

We searched through six main online news portals of Indonesia (Kompas.com, tempo.com, antaranews.com, detik.com, cnbcindonesia.com, and cnnindonesia.com) from 29th to 30th January 2024. The keywords used included “breastmilk donor,” “expressed breastmilk,” and “wet nursing” in the Indonesian language, Bahasa Indonesia. The term donor human milk is defined as expressed breastmilk that is voluntarily donated by a mother directly to babies other than her child, as well as the practice of wet nursing (cross-nursing). Articles published from 1 January 2018 to 31 December 2023 were included because this period was considered to represent the situation before, during, and after the COVID-19 pandemic. Articles published outside Indonesia were excluded.

**Figure 1:**
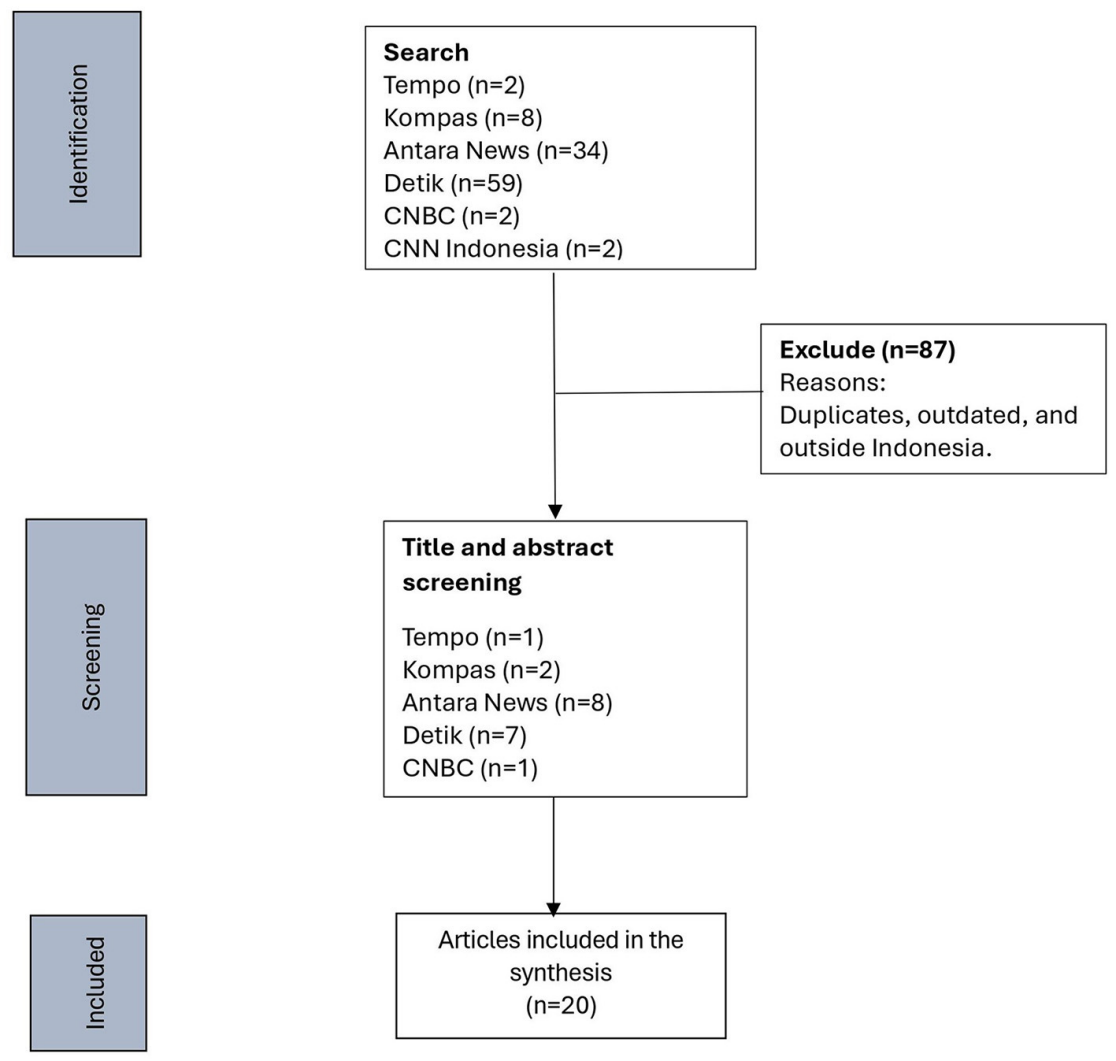
Search flowchart.

A total of 107 articles were found from the six online news portals, but only 20 articles were included for analysis using a qualitative media content analysis approach (17). News media was chosen for its significant impact and effects on public awareness, perceptions, and, sometimes, behavior (17). We employed a qualitative content analysis (18), which, in contrast to quantitative content analysis, is not an automatic process of counting manifest text elements but instead requires an in-depth study. The qualitative content analysis can be either inductive or deductive, and we analyzed the data inductively (19). The full process of the search and selection is described in Figure 1. The collected articles were recorded in an Excel sheet. Both authors read the full articles repeatedly and coded data individually. These articles were discussed, amended, and refined as a team. The potential themes were discussed and then agreed upon (Table 1). The details of the included articles are in Table 2.

**Table 1:** Data analysis structure.

**Theme**	**Definition**	**Code**	**Definition**
National and religion-based regulation	Articles contain the regulations required as the basic guide for safe access for donor human milk process	National regulation	Articles contain regulation issued by the authorized government of Indonesian republic as the basic guide on safe access for DHM
		Islamic law	Articles mention religious regulation binding for Muslim, based on the verses in Al Quran, or sunnah from holy prophet's role model. The law are major references for Muslim community in daily life practice
The whys and wherefores of donor milk	Situation and time settings where the article on donor human milk were published	COVID-19 pandemic	The global pandemic happens rapidly, disrupt the maternity care, including in labor process where mother and infant was separated and unable to breastfeed. This condition led to high demand for DHM described in the included article
		World breastfeeding week	World Breastfeeding Week, which is celebrated in the first week of August, is a period of time where DHM is intensively promoted as part of breastfeeding alternative option, through the media in Indonesia, including by the Government
		Disaster or emergency situation	Mothers and infants who were impacted by disaster, need breastfeeding supports, including access to DHM
		General situation	News on DHM is also being published when it draws public attention such as involving celebrities, criminal act, and others
Recommendations from authorized organizations, healthcare professionals and Islamic scholar	The best course of action put by experts in their fields, from health area up to religious scholar	WHO	WHO recommendation on infant and young child feeding stated that breastfeeding as the golden standard
		Indonesian Pediatric Society (IDAI)	As the professional association for pediatrics in Indonesia, IDAI provides recommendation on breastfeeding promotion, protection, and support nationally in Indonesia
		Indonesian Breastfeeding Mothers Association (AIMI)	AIMI is a national peak body for peer support in breastfeeding promotion, support and advocacy that has many branches in many provinces in Indonesia provides recommendation on DHM practice in the community
		Islamic scholar	As Indonesia is a Muslim-majority country, Islamic scholar provides explanation on the practice of DHM based on the Quran (the Islamic bible)
Contradict feelings of mothers	The conflicting feelings and emotions of mothers who intend to donor breastmilk or access donor breastmilk	Willingness to help/generosity	Mothers donated their expressed breastmilk or wet-nurse others' babies because they want to help other mothers who have challenges in breastfeeding their babies or help babies who lost their mothers due to various reasons
		Concern/anxiety	Despite acknowledging the benefit of breastfeeding, mothers had concern for the security of the DHM and/or feeling confused on how to access DHM
Negative impact from the lack of national regulation	Adverse effect that occurs due to the shortfall of national regulation regarding DHM	Misuse/sexual assault	Incidents where a male perpetrator was pretending to be a father who was looking for donor human milk and then sexually assaulted mothers who intended to help him
		Worry feeling from mothers	Mothers feel worried on how to access DHM and of its security, which then leads to formula feeding

**Table 2:** Included articles.

**No**.	**News portal**	**Publication date**	**Title**	**Link**	**Codes**	**Themes**
1.	Tempo.co	10 August 2021	Cerita Mona Ratuliu Menyusui Dua Anak, Pertama Kali Diantre Bayi-bayi (33)	https://cantik.tempo.co/read/1492841/cerita-mona-ratuliu-menyusui-dua-anak-pertama-kali-diantre-bayi-bayi	wet-nursing, mother died, tandem nursing, WBW	The whys and wherefores of donor human milk (Theme 1)
2.	Kompas.com	12 August 2021	Permintaan Donasi ASI Meningkat Lebih dari Dua Kali Lipat, Sebagian karena Pandemi (24)	https://megapolitan.kompas.com/read/2021/08/12/21351301/permintaan-donasi-asi-meningkat-lebih-dari-dua-kali-lipat-sebagian-karena	Non-government organization, milk-sharing platform, pandemic, increasing demand	The whys and wherefores of donor human milk (Theme 1)
3.	Kompas.com	2 August 2021	Donor ASI Bermunculan Saat Banyak Ibu Meninggal karena Covid-19, Ini Pro dan Kontranya (21)	https://megapolitan.kompas.com/read/2021/08/02/15051591/donor-asi-bermunculan-saat-banyak-ibu-meninggal-karena-covid-19-ini-pro	pandemic, benefits, concern for health risk, social media, milk screening procedure	The whys and wherefores of donor human milk (Theme 1); Negative impact from the lack of national regulation (Theme 4)
4.	Antaranews.com	14 September 2021	Mama muda rajin donorkan ASI-nya di Panti Asuhan Surabaya (31)	https://www.antaranews.com/berita/2391957/mama-muda-rajin-donarkan-asi-nya-di-panti-asuhan-surabaya	personal donation, informal breastmilk sharing, excessive breastmilk production, orphanage	The whys and wherefores of donor human milk (Theme 1)
5.	Antaranews.com	5 August 2021	IDAI minta Pemerintah segera buat aturan terkait donor ASI (34)	https://www.antaranews.com/berita/2310222/idai-minta-pemerintah-segera-buat-aturan-terkait-donor-asi	regulation, healthcare professional association, WBW, milk bank, procedure, screening	The whys and wherefores of donor human milk (Theme 1)
6.	Antaranews.com	6 August 2021	ASI ibu positif COVID-19 tidak tularkan virus kepada bayi (28)	https://www.antaranews.com/berita/2311226/asi-ibu-positif-covid-19-tidak-tularkan-virus-kepada-bayi	pandemic, alternative feeding option, lactation consultant	The whys and wherefores of donor human milk (Theme 1); Recommendations from authorized organizations, healthcare professionals and Islamic scholars (Theme 3)
7.	Antaranews.com	5 August 2021	Kemenkes kampanyekan pemberian ASI melalui Pekan Menyusui Sedunia 2021 (29)	https://www.antaranews.com/berita/2310294/kemenkes-kampanyekan-pemberian-asi-melalui-pekan-menyusui-sedunia-2021	pandemic, alternative feeding option, ministry of health, WBW	The whys and wherefores of donor human milk (Theme 1); Recommendations from authorized organizations, healthcare professionals and Islamic scholars (Theme 3)
8.	Antaranews.com	12 August 2021	IDAI: Ibu yang terinfeksi COVID-19 tetap bisa menyusui bayinya (26)	https://www.antaranews.com/berita/2322954/idai-ibu-yang-terinfeksi-covid-19-tetap-bisa-menyusui-bayinya	pandemic, healthcare professional association, alternative feeding option, infected mother	The whys and wherefores of donor human milk (Theme 1); Recommendations from authorized organizations, healthcare professionals and Islamic scholars (Theme 3)
9.	Antaranews.com	3 August 2021	Amankah obat COVID-19 diminum ibu menyusui yang positif? (25)	https://www.antaranews.com/berita/2304282/amankah-obat-covid-19-diminum-ibu-menyusui-yang-positif	pandemic, medicine, alternative feeding option, milk donor screening, feeding media, infected mother	The whys and wherefores of donor human milk (Theme 1); Recommendations from authorized organizations, healthcare professionals and Islamic scholars (Theme 3)
10.	Antaranews.com	6 November 2020	Ibu positif COVID-19 bisa tetap menyusui secara aman (27)	https://kalsel.antaranews.com/berita/211720/ibu-positif-covid-bisa-tetap-menyusui-secara-aman	pandemic, alternative feeding option, milk donor screening, feeding media, infected mother	The whys and wherefores of donor human milk (Theme 1); Recommendations from authorized organizations, healthcare professionals and Islamic scholars (Theme 3)
11.	Antaranews.com	12 August 2018	Ditinggal wafat ibunda karena gempa, bayi Alfia butuh ASI (30)	https://www.antaranews.com/berita/736211/ditinggal-wafat-ibunda-karena-gempa-bayi-alfia-butuh-asi	earthquake, disaster, orphaned baby, alternative feeding option, supply	The whys and wherefores of donor human milk (Theme 1)
12.	Detik.com	3 August 2021	Fenomena Donor Asi: Banyak Ibu Meninggal Akibat COVID-19 (23)	https://news.detik.com/berita/d-5667166/fenomena-donor-asi-banyak-ibu-meninggal-akibat-covid-19	mother died of covid	The whys and wherefores of donor human milk (Theme 1)
13.	Detik.com	12 August 2023	Viral Pria Fetish ASI, Ibu Wajib Perhatikan Ini Sebelum Memberi Donor ASI (40)	https://health.detik.com/berita-detikhealth/d-6872752/viral-pria-fetish-asi-ibu-wajib-perhatikan-ini-sebelum-memberi-donor-asi	sexual assault, alternative feeding option, regulation, informal milk sharing, worry	Negative impact from the lack of national regulation (Theme 4); Contradict feelings of mothers (Theme 5)
14.	Detik.com	25 February 2022	Bayi Boleh Diberikan Susu dari Donor ASI, Ini Dalil dan Penjelasannya (36)	https://food.detik.com/info-kuliner/d-5958021/bayi-boleh-diberikan-susu-dari-donor-asi-ini-dalil-dan-penjelasannya	Islamic rules, milk bank, donor background record	National and religion-based regulation (Theme 2); Recommendations from authorized organizations, healthcare professionals and Islamic scholars (Theme 3)
15.	Detik.com	7 September 2022	Kata Ustazah: Mau Donor ASI? Perhatikan Hal Ini Berkaitan dengan Mahram (35)	https://hot.detik.com/celeb/d-6276985/kata-ustazah-mau-donor-asi-perhatikan-hal-ini-berkaitan-dengan-mahram	Islamic rules, mahram, marriage	Recommendations from authorized organizations, healthcare professionals and Islamic scholars (Theme 3)
16.	Detik.com	12 August 2023	Tipu-tipu Pria Fetish ASI: Ngaku Cari Donor ASIP-Tak Mampu Beli Pompa ASI (39)	https://health.detik.com/berita-detikhealth/d-6872362/tipu-tipu-pria-fetish-asi-ngaku-cari-donor-asip-tak-mampu-beli-pompa-asi	lactophilia, sexual assault, feelings	Recommendations from authorized organizations, healthcare professionals and Islamic scholars (Theme 3); Negative impact from the lack of national regulation (Theme 4); Contradict feelings of mothers (Theme 5)
17.	Detik.com	13 August 2023	Heboh Wanita Jadi Korban Pria Fetish ASI, Begini Modus Pelaku (41)	https://health.detik.com/berita-detikhealth/d-6873252/heboh-wanita-jadi-korban-pria-fetish-asi-begini-modus-pelaku	sexual assault, regulation, AIMI response, hospital, premature baby	Negative impact from the lack of national regulation (Theme 4); Contradict feelings of mothers (Theme 5)
18.	Kompas.com	14 July 2021	Sebelum Istri Meninggal Dunia, Chiko Hakim Cari Donor ASI (32)	https://www.kompas.com/hype/read/2021/07/14/162647966/sebelum-istri-meninggal-dunia-chico-hakim-sempat-cari-donasi-asi-buat	celebrity, orphaned baby, donor request	The whys and wherefores of donor human milk (Theme 1)
19.	CnbcIndonesia.com	13 May 2020	Ibu Menyusui Positif Covid-19, Apakah Bisa Memberikan ASI? (22)	https://www.cnbcindonesia.com/market/20200513115222-17-158134/ibu-menyusui-positif-covid-19-apakah-bisa-memberikan-asi	pandemic, WHO, alternative feeding option, infected mother, benefits of breastfeeding	The whys and wherefores of donor human milk (Theme 1); Recommendations from authorized organizations, healthcare professionals and Islamic scholars (Theme 3)
20.	CNNindonesia.com	21 August 2018	Kriteria Tepat MPASI untuk Cegah Anak Gagal Tumbuh (38)	https://www.cnnindonesia.com/gaya-hidup/20180814131158-255-322130/kriteria-tepat-mpasi-untuk-cegah-anak-gagal-tumbuh	stunting prevention, complementary feeding, breastmilk composition, infant formula, pediatric	Recommendations from authorized organizations, healthcare professionals and Islamic scholars (Theme 3)

The trustworthiness of the content analysis was evaluated using Lincoln and Guba's criteria, which included the following (20): credibility, transferability, dependability, and confirmability. Credibility was achieved by describing the data analysis process in sufficient detail and through a multidisciplinary investigator collaboration, which involved a hospital-based qualified breastfeeding counselor (AH), an International Board Certified Lactation Consultant, and a university-based researcher (AP). Both authors were Indonesian, were members of the board committee of the Indonesian Breastfeeding Mothers Association (*Asosiasi Ibu Menyusui Indonesia/*hereafter referred to as AIMI), and have a public health background. This study's findings might be transferrable to other low- and middle-income countries (LMICs) having similarities to Indonesia; however, it cannot be guaranteed that the results are generalizable. To demonstrate dependability, all the raw data and analysis processes were documented to provide an audit trail. Confirmability was established through multiple team meetings discussing the analysis.

## 3 Results

We identified the following five themes: (1) The whys and wherefores of donor human milk, (2) national and religion-based regulation, (3) recommendations from authorized organizations, healthcare professionals, and Islamic scholars, (4) the negative impact from the lack of national regulations, and (5) contradictory feelings among mothers.

### 3.1 Theme 1: the whys and wherefores of donor human milk

Most of the studies were published at the time of the COVID-19 pandemic (21–28). Despite acknowledging the benefits of breastmilk, many people were worried about the possibility of transmitting the SARS-CoV-2 virus by giving breastmilk through direct breastfeeding, by giving expressed breastmilk from own mother, or by giving donated expressed breastmilk. The studies discussed whether consuming donor human milk was safe during the pandemic.

Some studies discussing donor human milk were published during World Breastfeeding Week, which is celebrated every August from the 1st to the 7th (29). Donor human milk was recommended by the Indonesian Pediatric Society as an alternative solution when mothers cannot breastfeed their babies.

One study shared the need for DHM during an emergency, specifically an earthquake (30). It discussed a case where the emergency response team looked for and successfully found donor human milk for an orphaned baby.

Three studies described the experience of two Indonesian celebrities and one social media celebrity (commonly referred to as *celebgram*) (31–33). One was a male celebrity whose wife died, and he stated his intention to look for donor milk. The other one was a female celebrity who became a wet nurse mother for her nephew, whose mother died after birth. The *celebgram*'s story primarily concerned her experience in donating expressed breastmilk to babies in orphanages.

### 3.2 Theme 2: national and religion-based regulation

In one study, the Indonesian Pediatric Society (*Ikatan Dokter Anak Indonesia*—hereafter referred to as IDAI) and AIMI requested the Indonesian government to establish a clear and strong regulation regarding DHM (34). This recommendation is due to the reality of informal human milk sharing in the community, which involves the increasing request for expressed breast milk through social media without a reliable screening process. As the largest breastfeeding support group in Indonesia, AIMI receives information about many mothers and families who require DHM but have no access to it.

In addition, some articles discussed religion-based regulations, namely Islamic law, regarding providing or accessing DHM (35, 36). There are rules regarding DHM, for instance, infants who receive DHM, both directly through wet nursing and from expressed breastmilk for several times until the infant is satiated, are considered the donor's milk daughter or son, and this child's relationships with the donor's biological children become those of milk siblings, which means that marriage is forbidden between them (37).

### 3.3 Theme 3: recommendations from authorized organizations, healthcare professionals, and Islamic scholars

Many of the studies mentioned recommendations regarding donor human milk from authorized organizations, such as AIMI and IDAI (22, 26–29, 38). They explained the best practices concerning DHM, which included the screening procedure. Furthermore, they also suggested the development of a clear regulation to reduce the health risks of unscreened donor milk.

As a Muslim-majority country, there were also recommendations from Islamic scholars based on the Quran, the holy book of Islam (35, 36). The practice of DHM is also regulated in the Quran, including the implication, such as when the recipient baby and the donor mother and her biological child(ren) become family, then marriage among the children would be considered permanently unlawful (*mahram*).

### 3.4 Theme 4: negative impact from the lack of national regulations

In some studies, healthcare professionals, authorized organizations, and Islamic scholars recommended some best practices as they believed that national regulations regarding donor human milk were lacking (34, 39, 40). The lack of national regulations has led to varied informal practices occurring in society, which are unregulated and risky for the mother, such as scams and assaults (39–41).

Furthermore, the IDAI has urged the Indonesian government to issue clear regulations to establish a legal framework for DHM and to ensure that safe and quality DHM is provided based on medical indications.

### 3.5 Theme 5: contradictory feelings among mothers

Some studies mentioned the motivation and feelings of donor mothers, such as the intention to help other mothers, regardless of not knowing the recipient mothers/babies (31, 33).

Three studies discussed the misuse of donor human milk, reporting incidents where a male perpetrator, pretending to be a father, sexually assaulted mothers who intended to help him (39–41). This incident made headlines in the news for some time and made a lot of mothers feel worried and anxious regarding donating their expressed breastmilk.

## 4 Discussion

The Indonesian government protects, supports, and promotes breastfeeding and has launched several national regulations in this regard, including Government Regulation No. 33/2012 on Breastfeeding regarding exclusive breastfeeding (10), which has recently been updated to Government Regulation No. 28/2024 (42). This regulation was developed as a guideline for the Law of Health No. 36/2009 (43), which was later revised as the Omnibus Health Law (Law No. 17/2023) (44). This regulation commands every mother to exclusively breastfeed their baby for the first 6 months and to continue until 2 years. Article 11 of the regulation states that when a baby's own mother cannot provide exclusive breastmilk to the baby, it can be provided by a donor mother. However, there are several requirements for donor human milk:

a. Requests must be made by the baby's own mother or family.
b. The identity, religion, and address of the donor mother must be clearly identified by the recipient baby's own mother or her family.
c. Agreement must be obtained from the donor mother after knowing the identity of the recipient baby.
d. The donor mother must be in good health and must not have medical issues, which include being HIV-negative, not having HSV-1 infection in the breast region, not undergoing anti-epileptic or opioid therapy, not having consumed radioactive iodine-131 within 2 months, using numerous topical iodine, such as povidone–iodine, or not receiving cytotoxic chemotherapy.
e. Donor human milk (DHM) must not be for sale.

However, all the regulations mentioning DHM only acknowledge it as an alternative infant feeding method when breastfeeding is not an option. There are no detailed procedures describing how mothers and families can access DHM or how mothers can donate their expressed breast milk. A study mentioned the recommendation from the IDAI that human donor milk should be prioritized for those who are really in need, such as premature or sick babies, or those who are separated from their mothers, rather than for mothers with perceived low supply issues, who possibly need breastfeeding support. Perceived low milk supply is experienced by many mothers and is one of the factors that influence early cessation of breastfeeding (45). The IDAI's recommendation aligns with the Australian Breastfeeding Association (ABA)'s policy, which encourages using donor milk that supports rather than replaces breastfeeding (46).

According to the data at the time of writing, Indonesia does not have a human milk bank, and hence, the practice of donor human milk occurs informally in the community. This practice is due to the generosity and willingness of mothers to help other mothers and babies (47, 48). The strong belief that breastfeeding the offspring is mandatory, which is based on the Quran, has influenced most Indonesian women to consider breastfeeding the norm. A majority of Indonesian people are Muslims (87%) (49), and breastfeeding is recommended as the best practice and is mentioned as one of the parents' responsibilities in the Quran. For example, it is the father's responsibility to ensure his baby is getting the best nutrition by being breastfed; therefore, he needs to fulfill the wife's need to be able to breastfeed. If it cannot be achieved, then the father is responsible for finding a wet nurse or DHM. Many Islamic scholars in Indonesia also provide recommendations on how to fulfill the child's right to be breastfed as regulated in the Quran (50).

The development of a human milk bank in Indonesia should consider the Islamic regulation (*syariat*), in addition to the health regulation. The Fatwa from the Council of Indonesian Ulama regulates kinship for DHM (51) and recommends that the Indonesian Ministry of Health issue a regulation on DHM based on the Fatwa. Singapore successfully established a human milk bank through the engagement of the relevant authorities and open dialogue. Finding common ground may allow vulnerable preterm infants to benefit from DHM in a manner that respects and does not compromise religious beliefs (52). A Malaysian study highlighted that the establishment of a religiously abiding human milk bank is achievable by educating mothers on breastfeeding benefits and addressing their religious concerns (53). Furthermore, the attitude and behavior of healthcare professionals also influenced the development of a human milk bank in one public hospital in Surabaya, Indonesia (54).

As a part of an informal sharing method, recommendations to ensure the safety of DHM should emphasize the importance of medical screening of the donor and safe milk handling practices (55). The IDAI has a guideline regarding DHM for public use to minimize the risk of mishandling donated expressed breastmilk (56). As a series of medical tests that are required is not covered by any health insurance, the medical screening for safety is mostly done by interview alone and is based on trust. This might bring a greater risk of a lack of reliability. As mothers' and families' knowledge of the benefits of breastfeeding is improving, the demand for DHM is also increasing. Therefore, the development of an official human milk bank is becoming more important. Many informal online groups on the Internet and social media have been established to bridge the demand for and supply of DHM. This is understandable as Indonesia has a high percentage of users of social media, such as Facebook, Instagram, and WhatsApp (15, 57). Although it was a rare case, the incident where a man pretended to be a father searching for DHM for his child and approached a potential donor to abuse her is an example of the negative impact of the lack of national regulations. Therefore, the IDAI also recommended that the government establish a formal organization or authority to regulate this matter (56, 58).

The use of DHM in emergency situations is already mentioned in the Guideline of Nutrition in Disaster Management published by the Indonesian Ministry of Health (59); however, the guideline only mentions the requirements adopted from the Government Regulation No. 33/2012 (10), which is now updated to the Government Regulation No. 28/2024 (42). The guideline does not describe how and where mothers can access DHM. During disasters in Indonesia, formula donation commonly occurs (60–62). This practice is due to the regulation that was issued by the local government that allows formula donations to take place with the approval from the head of the local health office (63, 64). This practice has been shown to increase the incidence rate of diarrhea among infants and young children who received the donated breastmilk substitute (61). Globally, the challenges in maintaining breastfeeding during an emergency, especially with formula donation, have been shown in many studies (65–69).

Eight out of the 20 articles included in this study were published during the COVID-19 pandemic (21–23, 25–28). Although the WHO stated that COVID-19-infected mothers could continue breastfeeding with a safe protocol (70), many maternity services implemented protocols that disrupted the initiation of breastfeeding (71–77). Furthermore, guidelines published by various authorities and countries were inconsistent (78). However, as the pandemic progressed, many studies showed that breastfeeding both directly and via expressed breastmilk provided significant benefits to the infants (79). As highlighted in the included studies in this study (21–24), many families searched for DHM when the mothers were infected with COVID-19. Noting this, AIMI and IDAI have recommended the development of a human milk bank.

To the best of our knowledge, our study is the first to examine the experience of DHM in Indonesia using a qualitative media content analysis approach. The use of online media is making it possible to capture what is really happening in the community as online media is rapidly growing in Indonesia. This study has limitations, for example, we limited the search to the top six online news portals within the past 5 years. We did not search through other media, such as newspapers and magazines; this may have narrowed the results. Furthermore, after the screening of the title and full text, the total number of articles included was only 20, which might not be considered a large sample. However, the five-year period was considered sufficient for capturing the experience within a society as the regulations and conditions were not much different from the current ones and it represented the period of time before, during, and after the COVID-19 pandemic.

## 5 Conclusion

The Indonesian government protects, supports, and promotes breastfeeding as regulated in Government Regulation Number 33 Year 2012, later updated to Government Regulation Number 28 Year 2024, which includes the use of DHM. However, due to the lack of detailed information on how to access or donate expressed human milk and the absence of a human milk bank in place, informal human milk sharing is inevitably occurring in the community. This also has raised concerns among authorized organizations, healthcare professionals, and Islamic scholars.

Consequently, there are negative impacts experienced by mothers, both the donor and recipient, including contradictory feelings, from the lack of a clear regulation regarding DHM and the lack of development of a human milk bank.

Engaging with Islamic scholars and healthcare professionals to develop clear guidelines and regulations to enable mothers' and families' access or contribution to DHM in a safe and accountable way is critical for preventing the occurrence of further problems in Indonesian society.

## Data Availability

The raw data supporting the conclusions of this article will be made available by the authors, without undue reservation.
